# Indirect immunofluorescence on rat bladder epithelium in patients with pemphigus vulgaris with an extended follow‐up

**DOI:** 10.1002/ski2.142

**Published:** 2022-06-21

**Authors:** Mahsa Samadi, Anahita Najafi, Amir Naziriyan, Roja Toosi, Atefeh Faramarzi, Kamran Balighi, Pedram Noormohammadpour, Hamidreza Mahmoudi, Maryam Daneshpazhooh

**Affiliations:** ^1^ School of Medicine Tehran University of Medical Sciences Tehran Iran; ^2^ Department of Dermatology Autoimmune Bullous Diseases Research Center Tehran University of Medical Sciences Tehran Iran

## Abstract

**Background:**

Indirect immunofluorescence (IIF) on rat bladder epithelium (RBE) has been widely used to detect anti‐plakin antibodies present in paraneoplastic pemphigus (PNP). However, anti‐plakin antibodies have also been found in a group of patients with pemphigus vulgaris (PV).

**Objectives:**

To assess the reactivity rate of PV sera in IIF using RBE as substrate and the diagnostic usefulness of the aforementioned test.

**Methods:**

Patients diagnosed with PV presenting to Razi Hospital, Tehran, Iran, were recruited. The patients' demographics, disease severity, and response to the initial treatment were recorded. Sera were collected and tested by IIF on RBE and by desmoglein 3/1 (Dsg 3/1) enzyme‐linked immunosorbent assay. Patients were followed up closely for a mean of 53.9 months for any evidence of malignancy.

**Results:**

Forty‐six patients were enroled (mean age of 42.9 years old, 31 females). Nine sera (19.6%) showed reactivity in IIF on RBE. Mean serum anti‐Dsg levels did not differ significantly among the two groups with positive and negative IIF results. Negative anti‐Dsg3 was related to a higher positive rate in IIF on RBE. There was no significant correlation between the reactivity of IIF on RBE and patients' demographic, clinical, or serological characteristics.

**Conclusions:**

IIF on RBE is a sensitive test for detecting antibodies against plakins. However, it has a relatively high false‐positive rate in PV, probably due to the epitope spreading phenomenon. This test should be suggested when there is a clinical or immunohistopathological suspicion of PNP and should be interpreted with caution.

## Graphical abstract

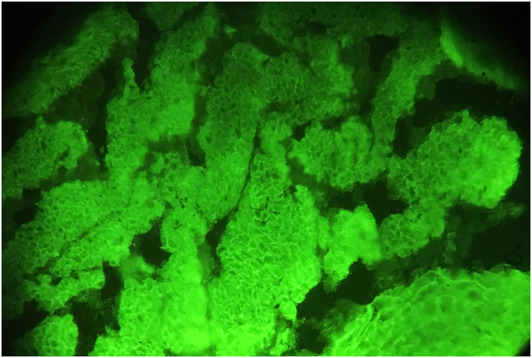

1

**What is already known about this topic?**

- The Indirect immunofluorescence (IIF) test on rat bladder epithelium (IIF‐RBE) has been known as a helpful diagnostic method to discriminate patients with paraneoplastic pemphigus (PNP) from patients with pemphigus vulgaris (PV). Although other diagnostic methods have been introduced for this matter, this test has more global accessibility and feasibility. However, the reported specificity values for this test in different studies have been discrepant. Moreover, they had a short‐term follow‐up. Therefore, interpretation of the IIF‐RBE results has been quite challenging in clinical settings.

**What does this study add?**

- We evaluated the reactivity rate of PV sera in the IIF test on RBE and the diagnostic usefulness of the aforementioned test. Extended close follow‐up was performed for any evidence of malignancy to support our findings on the diagnostic validity of this test. Our findings can help practitioners implement this test and interpret the results in a more cautious manner.

## 2 BACKGROUND

Pemphigus refers to a wide range of mucocutaneous (MCPV) autoimmune bullous diseases (AIBD) characterised by Ig G‐mediated acantholysis, leading to intraepithelial blisters and erosions. Three major types of pemphigus are known: PV, pemphigus foliaceus (PF), and PNP.^1^ PV is characterised by mucosal and/or skin blisters clinically, and antibodies targeting desmoglein (Dsg) 3 and to a lesser extent, Dsg 1. Paraneoplastic pemphigus is a rare polymorphous autoimmune blistering disease of the skin and mucus membranes accompanied by neoplasms. The most common presentation of PNP is persistent oral lesions, followed by polymorphic cutaneous manifestations that can mimic other skin diseases such as PV.

Diagnosis of PNP is made based on clinical signs, histopathology, and immunological features. Suprabasal acantholysis and interface dermatitis are common histopathological findings. Direct immunofluorescence (DIF) typically shows IgG and complement deposits in intercellular spaces in the epidermis and in some cases, in the basement membrane zone. The presence of autoantibodies against the plakin family is a characteristic feature of PNP.^2^, ^3^, ^4^ Patients with PNP have a 5‐year survival rate of 38%. Therefore, early diagnosis is crucial and leads to better treatment outcomes. Anhalt et al. first described IIF testing on RBE as a feasible screening method for PNP since various plakins are expressed in RBE.^5^ If other specific detection methods (immunoblot, immunoprecipitation (IP), ELISA) are not available, evaluating anti‐plakin antibodies on IIF‐RBE may be helpful for diagnosis.^6^, ^7^ Most studies on IIF‐RBE have shown a high sensitivity and specificity for the diagnosis of PNP.^8^, ^9^, ^10^ However, the autoantibodies directed against the plakin family have been found in other skin conditions such as PV and PF, thus reducing the specificity of IIF‐RBE for PNP, making the diagnosis quite challenging.^7^, ^8^, ^11^

Our study aimed to assess IIF‐RBE diagnostic usefulness by testing PV sera. We also examined different disease aspects among PV cases with true‐negative and false‐positive IIF‐RBE results. We evaluated any possible correlation between IIF‐RBE results and the severity of the disease. We also performed a thorough examination to find any possible underlying malignancy in patients with PV and a positive IIF‐RBE and followed patients to see if they later developed any neoplasm.

## 3 METHODS

### 3.1 Patient selection

We enroled consecutive patients, newly diagnosed with PV, presenting to Razi Hospital, a tertiary referral centre for skin diseases in Tehran, Iran, between March 2016 and March 2018. The diagnosis was confirmed upon typical clinical features with histopathological findings of suprabasal acantholysis and intercellular IgG and/or C3 deposits on DIF. None of the selected patients had a history of associated neoplasm, and all had initially received systemic prednisolone 0.5–1 mg/kg and a total dose of 2 g of rituximab. The disease severity was measured using Pemphigus Disease Area Index (PDAI) score. The response to the initial treatment was classified according to the generally accepted criteria.^12^ Sera were collected from patients after obtaining written informed consent and were tested. Test results were recorded as positive or negative in blinded readings. Patients were followed up until March 2021 for a mean of 53.9 months (SD: 9.9). None of the participants were diagnosed with any malignancies until the completion of this study. The study was approved by the ethics committee of Tehran University of Medical Sciences.

### 3.2 Indirect immunofluorescence on rat bladder epithelium (indirect immunofluorescence test on rat bladder epithelium)

The IIF testing was conducted according to previously established methods.^5^, ^13^ All sera were serially diluted from 1:10 to 1:160 in phosphate‐buffered saline (PBS). Cryostat sections of RBE were used as substrate. Sections were washed with PBS and then incubated with diluted sera for 30 min. After the second PBS washing, sections were covered with fluorescein isothiocyanate‐conjugated rabbit antihuman IgG (DAKO, Lustrum, Denmark) for another 30 min. After an additional PBS washing, the slides were set in buffered glycerol and examined under a fluorescence microscope. Titres of 1:40 or higher were considered positive.

### 3.3 Dsg1/3 enzyme‐linked immunosorbent assay (ELISA)

Serum anti‐Dsg1 and anti‐Dsg3 antibody values were measured using commercially available Dsg1 and Dsg3 ELISA kits (EUROIMMUN Medizinische Labordiagnostika AG, Lubeck, Germany). All serum samples were diluted 100‐fold as per the manufacturer's instructions. Data are expressed as units per millilitre (U/ml), and values above 20.0 U/ml are considered positive.

### 3.4 Statistical analysis

IBM SPSS Statistics for Windows, Version 26.0 (Armonk, NY: IBM Corp) was used to analyse the data. Parametric and non‐parametric continuous data were reported using mean (± standard deviation) and median (interquartile range), respectively. All continuous data were assessed for normality using the Shapiro‐Wilk normality test. Independent Samples *t*‐test and Mann‐Whitney *U* test were used for comparing means for parametric and medians for non‐parametric data. Indirect immunofluorescence test on rat bladder epithelium results were divided into positive and negative groups using 1:40 dilution as the cut‐off value. Pearson's chi‐square test was used to test for association between categorical data. Spearman rank correlation was used to assess the score relationship of IIF‐RBE results with serum anti‐Dsg1 and anti‐Dsg3 values, PDAI score, disease duration, disease phenotype, response to the initial treatment, age, and gender. A *p*‐value of less than 0.05 was considered significant.

## 4 RESULTS

Forty‐six patients were included in this study with a mean age of 42.9 (SD: 11.3), 31 of whom were females. The demographic and clinical data of the patients are presented in Table 1.

**TABLE 1:** The demographic and clinical data of patients with pemphigus vulgaris (PV)

Characteristic	Result
Age, years, mean ± SD (range)	42.9 ± 11.3 (22–69)
Gender, M/F	15/31
Phenotype, *n* (%)	
Mucosal	11 (23.9)
Mucocutaneous	34 (73.9)
Cutaneous	1 (2.2)
Response to the initial treatment, *n* (%)	
Remission	44 (95.7)
Active disease	2 (4.3)
Duration of disease, months^a^	3.0 (1.5–3.0), 1–18
PDAI score, of 250^a^	16.5 (6.5–33.15), 1–77
Follow up duration, months, mean ± SD (range)	53.9 ± 9.9 (36–74)

Abbreviations: F, female; M, male; n, number; PDAI, pemphigus disease area index; SD, standard deviation.

^a^ Data shown in median (quartile1‐quartile3), range.

**FIGURE 1:**
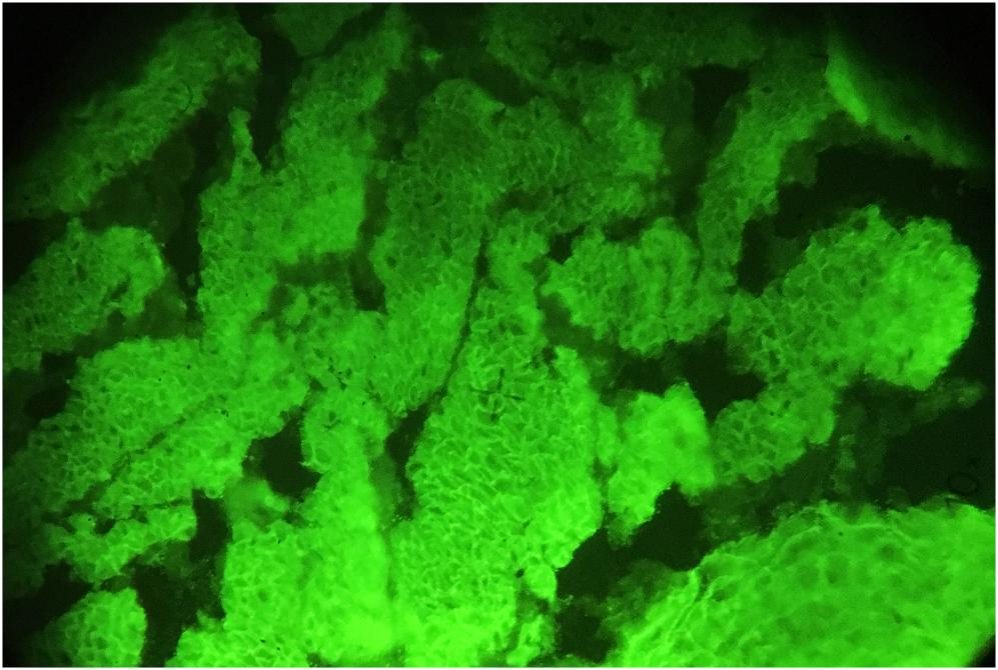
Reactivity of a patient's serum at the dilution of 1:40, diagnosed with pemphigus vulgaris (PV), on rat bladder epithelium (RBE); Revealed by fluorescein isothiocyanate (FITC) stain on Indirect immunofluorescence (IIF), under a fluorescence microscope, with 10‐times magnification

Among 46 PV sera, nine (19.6%) had positive IIF‐RBE results at the dilution of 1:40. At the dilution of 1:80, five (10.9%) sera still showed reactivity, and at the dilution of 1:160, only two (4.3%) sera were still positive (Figure 1). The antibodies were directed against the intercellular substance of RBE and belonged to the IgG class. Three (33.3%) of the IIF‐RBE positive sera belonged to patients with mucosal pemphigus vulgaris, and six (66.7%) of IIF‐RBE positive sera were from patients with a MCPV disease presentation. There was no statistically significant effect for phenotype on IIF‐RBE results. Data regarding IIF‐RBE test results and disease phenotype are shown in Table 2.

**TABLE 2:** IIF‐RBE versus disease phenotype^a^ ^,^ ^b^

	IIF‐RBE result	
Phenotype	Negative	Positive
MCPV	28 (82.4%)	6 (17.6%)
MPV	8 (72.7%)	3 (27.3%)
CPV	1 (100.0%)	0 (0.0%)
Total	37 (80.4%)	9 (19.6%)

Abbreviations: CPV, cutaneous pemphigus vulgaris; IIF‐RBE, indirect immunofluorescence on rat bladder epithelium; MCPV, mucocutaneous pemphigus vulgaris; MPV, mucosal pemphigus vulgaris.

^a^ Data shown as number of cases (% within phenotype group).

^b^ $X^{2}$ (1, 46) = 0.738, *P* = 0.691.

Values of the Dsg1 and Dsg3 ELISA results of two patients were missing. Out of 44 PV sera, using the manufacturer's suggested cut‐off value of 20.00 U/mL, the serum anti‐Dsg1 and anti‐Dsg3 ELISA were positive in 23 (52.3%) and 41 (93.2%), respectively. Patients whose sera were negative for anti‐Dsg3 had a significantly higher chance of obtaining a false‐positive result on IIF‐RBE, *p*‐value: 0.024. However, we found no statistically significant difference in mean serum anti‐Dsg1 and anti‐Dsg3 ELISA values among the patients with positive and negative IIF‐RBE test results. Table 3 presents the data of IIF‐RBE and anti‐Dsg ELISA results.

**TABLE 3:** IIF‐RBE versus anti‐Dsg1 and anti‐Dsg3

	IIF‐RBE result	
ELISA results	Positive	Negative
Anti Dsg‐1^b^ ^,^ ^a^		
Values, U/mL, mean ± SD	80.6 ± 80 0.4	59.9 ± 79.6
Positive	5 (21.7%)	18 (78.3%)
Negative	3 (14.3%)	18 (85.7%)
Anti Dsg‐3^c^ ^,^ ^a^		
Values, U/mL, mean ± SD	141.9 ± 115.6	171.3 ± 97.0
Positive	6 (14.6%)	35 (85.4%)
Negative	2 (66.7%)	1 (33.3%)

Abbreviations: Dsg, desmoglein; ELISA, enzyme‐linked immunosorbent assay; IIF‐RBE, indirect immunofluorescence on rat bladder epithelium; SD, standard deviation; U/mL, units per milliliter.

^a^ Data shown as number of cases (% within Dsg1/Dsg3 group).

^b^ $X^{2}$ (1, 44) = 0.410, *P* = 0.522.

^c^ $X^{2}$ (1, 44) = 5.088, *P* = 0.024.

Indirect immunofluorescence test on rat bladder epithelium test results had no statistically significant relationship with patients' demographics, disease phenotype, duration of disease, the severity of skin and mucous membranes involvement – based on PDAI score, response to the initial treatment, and serum anti‐Dsg1 and anti‐Dsg3 ELISA values. There was no significant correlation between serum anti‐Dsg1 and anti‐Dsg3 ELISA results and demographic and clinical characteristics.

## 5 DISCUSSION

Our results show that up to one‐fifth of PV sera carry autoantibodies against RBE antigens. Two out of nine patients whose IIF‐RBE results were positive had negative results for both anti‐Dsg one‐third, suggesting a possible subtype of patients with PV who carry autoantibodies against plakins but not against Dsgs.

Paraneoplastic pemphigus diagnosis can be challenging due to the significant overlap between clinical and histological features of PNP and other AIBD. Since PNP was introduced, serological assessments have been included in the diagnostic criteria. The associated autoantibodies have been explored using direct and indirect immunofluorescence, immunoblotting (IB), and IP techniques.^5^ IP detects autoantibodies with utmost specificity and remains the gold‐standard method.^8^, ^9^ However, the IP technique is costly, time‐consuming, highly skilled, and of limited availability in most world areas. Immunoblotting is a specific but technically‐demanding and time‐consuming technique that can only be performed in highly equipped laboratories. It also has a low sensitivity due to the denaturation of protein antigens during electrophoresis.^14^ In contrast to IP or IB techniques, immunofluorescence testing is feasible and widely accessible and has acceptable accuracy and has become the preferred screening method for PNP diagnosis.

The most frequently detected circulating autoantibodies in PNP target members of the plakin family, which include periplakin, envoplakin, desmoplakin (DP) 1 and 2, bullous pemphigoid antigen, and plectin.^15^, ^16^ Like PV and PF, patients with PNP might also express autoantibodies against Dsg1/3^6^, ^17^ Detection of auto‐antibody directed against envoplakin and periplakin is most specific, followed by DP1/2.^7^ Murine urothelium contains a high density of DP1/2, but not Dsg, envoplakin, or periplakin.^10^, ^18^ Indirect immunofluorescence on other tissues that contain desmoplakins has significant limitations, and no tissue is more reliable than RBE in diagnosing PNP.^10^ Therefore, RBE has become a suitable substrate for screening PNP.

Since introducing IIF‐RBE as a convenient screening tool for diagnosing PNP,^5^ researchers have examined its reliability in numerous studies. During the early investigations, IIF‐RBE was reported to be highly sensitive and specific for PNP. Nevertheless, those studies involved a limited number of cases. Liu et al. studied 17 PV sera, and only one (specificity of 94% among PV controls) was positive in IIF‐RBE.^19^ Later Helou et al. studied 28 patients with PNP and 29 patients with an unspecified autoimmune blistering disease with IIF on RBE and reported a specificity of 83%.^10^ Cozzani et al. found 21% positive IIF‐RBE while investigating autoantibodies in 48 PV sera, which is in accord with our results. They suggested a role for anti‐DP in disease severity.^20^ Ortolan et al. investigated 23 PV sera and found the overall reactivity of IIF‐RBE to be 22% in PV sera.^11^ Poot et al. enrolled 19 PNP and 24 PV participants in their study and found a 100% specificity for IIF‐RBE in PNP exclusion.^9^ The methodological differences in patient selection and assay performance should be considered when interpreting the discrepancies found in previous investigations. Our study revealed that however insignificant, most of the positive IIF‐RBE sera belonged to MCPV phenotype. Furthermore, we found no clear correlation between IIF‐RBE results with severity based on PDAI score and response to the initial treatment.

Our results and previous findings reinforce the principle of cautious interpretation of laboratory test results when making a diagnosis. Although screening by IIF‐RBE is a useful method in suspicious cases, a thorough clinical and histopathological correlation must be considered. Indirect immunofluorescence test on rat bladder epithelium should not be performed as a routine technique in patients with PV and should only be employed when there are suspicious clinical, histopathological, or DIF findings for PNP. The presence of autoantibodies against desmoplakins does not equate to a diagnosis of PNP. Plakin autoantibodies have been identified in other autoimmune skin conditions such as PV, PF, erythema multiforme, and toxic epidermal necrolysis.^8^, ^9^, ^20^, ^21^, ^22^ Therefore, a false‐positive IIF‐RBE in a fraction of cases with PV could be anticipated. A possible explanation for DP antibodies in PV is the epitope spreading phenomenon.^23^ DP1/2 link cytoskeleton intermediate filaments and desmosomal cadherins in the desmosome‐intermediate filament complex located in the cell membrane.^24^ Destruction of desmosomes during the disease course may introduce previously concealed antigens such as DP1/2 to the immune system, resulting in diverse autoantibodies. We suggest that DP1/2 may be considered as autoantigens for PV as well as PNP.

### 5.1 Limitations

Several limitations of this study warrant consideration. First, our study was carried out on relatively small sample size. Second, the patient sera were collected from a single recruitment site; therefore, the generalisability of our results to a larger population is debatable. Another limitation of our study is that the gold‐standard IP or IB techniques were not employed to exclude PNP due to their restricted availability. Therefore, PNP was ruled out based on the clinical features, typical histopathological findings, natural disease course, satisfactory response to conventional PV treatment, and a long malignancy‐free follow‐up. However, because more than 95% of cases with PNP present with a preexisting malignancy or develop a concomitant neoplasm soon after the onset of MCPV symptoms, the extended follow‐up of our cases minimises the chance of misdiagnosis.^4^, ^25^

## 6 CONCLUSION

Considering the relatively high false‐positive rate of IIF‐RBE in PV, this test should only be suggested whenever PNP is clinically or histopathologically compatible.

## AUTHOR CONTRIBUTIONS

**Mahsa Samadi**: Data curation (equal); Formal analysis (equal); Investigation (equal); Writing – original draft (equal). **Anahita Najafi**: Data curation (equal); Formal analysis (equal); Software (equal); Writing – original draft (equal). **Amir Naziriyan**: Data curation (equal); Formal analysis (equal); Investigation (equal); Writing – original draft (equal). **Roja Toosi**: Conceptualization (equal); Methodology (equal); Project administration; (supporting); Supervision (equal); Writing – review & editing (supporting). **Atefeh Faramarzi**: Conceptualization (equal); Methodology (equal); Project administration (supporting); Supervision (equal); Writing – review & editing (supporting). **Kamran Balighi**: Conceptualization (equal); Methodology (equal); Project administration (supporting); Writing – review & editing (supporting). **Pedram Noormohammadpour**: Conceptualization (equal); Methodology (equal); Project administration (supporting); Writing – review & editing (supporting). **Hamidreza Mahmoudi**: Conceptualization (equal); Methodology (equal); Project administration (supporting); Writing – review & editing (supporting). **Maryam Daneshpazhooh**: Conceptualization (equal); Methodology (equal); Project administration (lead); Supervision (equal); Validation (equal); Writing – review & editing (lead).

## CONFLICT OF INTEREST

The authors have no conflicts of interest to disclose.

## ETHICS STATEMENT

This study was conducted in accordance with the principles expressed in the World Medical Association Declaration of Helsinki, as revised in 2013. Written informed consent was obtained from the participants of this study. This study was approved by the ethics committee of Tehran University of Medical Sciences (Approval ID: IR.TUMS.MEDICINE.REC.1397.527).

## Data Availability

The datasets used and/or analyzed during this study are available on reasonable request from the corresponding author.
